# Protracted COVID-19 pneumonia in B-cell-depleted patients

**DOI:** 10.1093/rheumatology/keae703

**Published:** 2024-12-23

**Authors:** Orhan Efe, Gabriel Sauvage, James Chung, Anushya Jeyabalan, Ayman Al Jurdi, Harish S Seethapathy, Karen A Laliberte, John L Niles

**Affiliations:** Vasculitis and Glomerulonephritis Center, Massachusetts General Hospital, Boston, MA, USA; Division of Nephrology, Massachusetts General Hospital, Boston, MA, USA; Harvard Medical School, Boston, MA, USA; Vasculitis and Glomerulonephritis Center, Massachusetts General Hospital, Boston, MA, USA; Vasculitis and Glomerulonephritis Center, Massachusetts General Hospital, Boston, MA, USA; Vasculitis and Glomerulonephritis Center, Massachusetts General Hospital, Boston, MA, USA; Division of Nephrology, Massachusetts General Hospital, Boston, MA, USA; Harvard Medical School, Boston, MA, USA; Vasculitis and Glomerulonephritis Center, Massachusetts General Hospital, Boston, MA, USA; Division of Nephrology, Massachusetts General Hospital, Boston, MA, USA; Harvard Medical School, Boston, MA, USA; Vasculitis and Glomerulonephritis Center, Massachusetts General Hospital, Boston, MA, USA; Division of Nephrology, Massachusetts General Hospital, Boston, MA, USA; Harvard Medical School, Boston, MA, USA; Vasculitis and Glomerulonephritis Center, Massachusetts General Hospital, Boston, MA, USA; Vasculitis and Glomerulonephritis Center, Massachusetts General Hospital, Boston, MA, USA; Division of Nephrology, Massachusetts General Hospital, Boston, MA, USA; Harvard Medical School, Boston, MA, USA

Rheumatology key message

- B-cell-depleted patients carry a unique protracted COVID-19 risk, requiring strong suspicion and aggressive treatment.

Dear Editor, Given the wide use of vaccinations, the high prevalence of prior SARS-CoV-2 infections and the availability of anti-virals, complications from COVID-19 have dropped dramatically. Although SARS-CoV-2 infections remain prevalent, they are increasingly ignored or go unrecognized.

Meanwhile, B-cell depletion with anti-CD20 monoclonal antibodies (rituximab and others) has become a standard treatment for antibody-mediated autoimmune diseases, such as ANCA-associated vasculitis, systemic lupus erythematosus, multiple sclerosis, pemphigus vulgaris as well as for B cell malignancies. Full peripheral blood B-cell depletion typically lasts 6–12 months after each rituximab dose [1], completely blocks new antibody responses to SARS-CoV-2 infection and vaccines, and is associated with severe COVID-19 [2]. A lack of appreciation of the specific risk from B-cell depletion may result in a lack of recognition of severe COVID-19 cases.

We assessed the outcomes of SARS-CoV-2 infections among patients with partial or full B-cell depletion due to rituximab or other anti-CD20 monoclonal antibodies for autoimmune diseases at the Vasculitis and Glomerulonephritis Center at the Massachusetts General Hospital between 11 May 2023 and 14 June 2024 (See supplementary Data S1, available at *Rheumatology* online). The study was conducted in accordance with the Declaration of Helsinki and approved by the Institutional Review Board at Massachusetts General Hospital. Informed consent was waived because of the retrospective nature of the study.

Among 690 patients on B-cell depletion with anti-CD20 treatments, 83 (12%) had documented acute COVID-19. Among the 76 patients with sufficient follow-up data, 32% (24/76) had at least moderate COVID-19 [3] and 3% (2/76) died (Table 1).

**Table 1.** Clinical characteristics, treatments and outcomes of patients who develop protracted *vs* non-protracted COVID-19

	Non-protracted (*n* = 63)	Protracted (*n* = 13)	*P* value
Age, median (IQR)	63 (49–73)	60 (47–67)	0.8199^a^
Female, *n* (%)	42 (67)	7 (54)	0.465^b^
Race, *n* (%)			0.834^b^
White	56 (89)	12 (92)	
Black	2 (3)	1 (8)	
Asian	3 (5)	0 (0)	
Hispanic	2 (3)	0 (0)	
Primary disease, *n* (%)			
ANCA-associated vasculitis	40 (64)	8 (62)	
ANCA-negative vasculitis	4 (6)	0 (0)	
Eosinophilic granulomatosis with polyangiitis	2 (3)	1 (8)	
Systemic lupus erythematosus or lupus nephritis	4 (6)	3 (23)	
Minimal change disease or FSGS	4 (6)	0 (0)	
Others^c^	9 (14)	1 (8)	
Time from rituximab initiation to COVID-19 (years), median (IQR)	5.5 (2.3–8.4)	4.3 (2.1–7.2)	0.8623^a^
Time from last rituximab infusion to COVID-19 (months), median (IQR)	4.1 (1.6–10.0)	4.4 (1.7–7.7)	0.9395^a^
Patients with peripheral B cell count of <5/µL during active COVID-19 infection, *n* (%)	48 (76)	13 (100)	0.06^b^
Number of prior vaccinations, median (IQR)	4 (3–5)	2 (0–3)	0.3987^d^
Number of prior SARS-CoV-2 infections, median (IQR)	1 (0–1)	1 (0–1)	0.4910^a^
Serum anti-spike antibody titre at the time of COVID-19, U/mL (IQR) (*n* = 6 *vs* 6)	242 (25.6–1056)	1.4 (0–26.2)	0.0390^a^
Positive anti-nucleocapsid antibody at the time of COVID-19, *n* (%)	0/5 (0)	0/6 (0)	
Concurrent chronic Ig replacement, *n* (%)	6 (10)	0 (0)	
Initial course of treatment, *n* (%)			
Anti-viral	52 (83)	9 (69)	0.273^b^
Glucocorticoids	8 (13)	5 (38)	0.089^b^
IVIG	2 (3)	0 (0)	0.711^b^
Second course of treatment, *n* (%)			
Anti-viral	2 (3)	12 (92)	<0.001^b^
Glucocorticoids	3 (5)	11 (85)	<0.001^b^
IVIG	0 (0)	10 (77)	<0.001^b^
Disease severity,^e^*n* (%) [3]			<0.001^b^
Mild	52 (83)	0 (0)	
Moderate	7 (11)	5 (38)	
Severe	3 (5)	6 (46)	
Critical	0 (0)	1 (8)	
Death	1 (2)	1 (8)	
Durations of hospitalization (days), median (IQR)	0 (0–0)	10 (5–21)	<0.001^a^

a Wilcoxon rank-sum test.

b Fisher’s exact test.

c Others include polymyositis/dermatomyositis (*n* = 3), rheumatoid arthritis (*n* = 1), Sjogren’s disease (*n* = 1), membranous nephropathy (*n* = 2), anti-glomerular basement membrane disease (*n* = 1), fibrillary glomerulonephritis (*n* = 1) and undifferentiated connective tissue disorder (*n* = 1).

d Unpaired *t* test.

e A brief description of NIH 2024 Clinical Spectrum of SARS-CoV-2 Infection includes [3]:
*Asymptomatic or presymptomatic infection:* Individuals who test positive for SARS-CoV-2 using a virologic test but have no symptoms of COVID-19. *Mild illness:* Individuals who have signs and symptoms of COVID-19, such as fever, cough or sore throat, but do not have shortness of breath or abnormal chest imaging. *Moderate illness:* Individuals who show evidence of lower respiratory disease in clinical assessment or imaging and who have an oxygen saturation (SpO_2_) ≥94% on room air. *Severe illness:* Individuals who have a SpO_2_ <94% on room air, a ratio of arterial partial pressure of oxygen to fraction of inspired oxygen (PaO_2_/FiO_2_) <300 mmHg, a respiratory rate >30 breaths/min or lung infiltrates >50%. *Critical illness:* Individuals who have respiratory failure, septic shock or multiple organ dysfunction.

ANCA: anti-neutrophil cytoplasmic antibody; COVID-19: coronavirus disease; FSGS: focal segmental glomerulosclerosis; IQR: interquartile range; IVIG: intravenous immunoglobulin.

Strikingly, 13 of the 76 patients (17%) developed protracted COVID-19 pneumonia, which we defined as worsening disease after the 30-day mark based on clinical and radiologic assessment. These patients had more severe disease and prolonged hospitalizations. B cells were fully depleted at the time of their COVID-19 and their serum anti-SARS-Co-V2 spike and nucleocapsid antibodies were almost all undetectable (Table 1). Only one patient, who subsequently died, was on cytotoxic therapy, with azathioprine 50 mg daily. Recognition of ongoing COVID-19 was often delayed due to either lack of clinical suspicion or multiple negative nasal swabs. Computed tomography findings were often attributed to other aetiologies (Supplementary Fig. S1A–D, available at *Rheumatology* online). Outpatient testing of sputum samples for SARS-CoV-2 was usually refused due to the unavailability of a ‘validated’ assay for sputum. Meanwhile, in 38% (5/13), the diagnosis was only possible after testing of bronchoalveolar lavage or sputum samples.

A typical case was a 46-year-old female on B-cell depletion for lupus nephritis. Initially, she had mild COVID-19 and improved after remdesivir. Subsequently, she presented with pneumonia (Supplementary Fig. S1A, available at *Rheumatology* online) with multiple negative nasal swabs for COVID-19, was hospitalized twice, including intensive care, and received repeated antibiotics and high-dose steroids. The patient ultimately tested PCR positive for SARS-CoV-2 on bronchioalveolar lavage on day 35. Treatment with extended remdesivir and IVIG led to rapid recovery.

Patients with protracted or severe COVID-19 required repeated and extended courses of nirmatrelvir/ritonavir or remdesivir for up to 20 days. IVIG was also usually added in these cases, usually at a dose of 0.4 g/kg daily for 3–5 days (Table 1). IVIG treatment led to a sharp rise in serum anti-SARS-CoV-2 spike (Supplementary Fig. S1E, available at *Rheumatology* online) and total antibodies (Supplementary Fig. S1F, available at *Rheumatology* online) [4, 5]. Rapid clinical improvements ensued with treatment in all but one patient who died (Table 1). IVIG was delayed until the patient had advanced acute respiratory distress syndrome in the patient who died.

A unique risk of protracted COVID-19 exists in patients with B-cell depletion. Our observations suggest a few key points: (i) Serum SARS-CoV-2 anti-spike antibodies are virtually undetectable in patients who have been B-cell-depleted throughout their prior COVID-19 infections and vaccinations. (ii) Thus, B-cell depletion should trigger a high index of suspicion for protracted COVID-19. The risk for protracted COVID-19 was not appreciated by physicians due to prior vaccinations, recovery from prior COVID-19 and prolonged windows of improvement after initial antivirals. Although prior exposure to cytotoxic therapies during the pandemic may also have impacted the development of immunity against SARS-CoV-2, only one was on cytotoxic therapy at the onset of her COVID-19. (iii) Testing of lower respiratory tract samples and serial nasal swabs was often critical for diagnosis. (iv) All patients timely treated with the combination of antivirals with adjunctive IVIG improved rapidly. Our anecdotal experience suggests that adjunctive IVIG may provide benefit, which could be through the high level of neutralizing spike antibodies present in current IVIG preparations [4, 5], especially in patients with virtually undetectable serum anti-spike antibody titres to start.

In conclusion, understanding the risk, recognizing the pattern of disease, testing of sputum and bronchoscopy specimens and timely treatment are critical to control this outbreak of COVID-19 in patients with B-cell depletion.

## Supplementary Material

keae703_Supplementary_Data

## Data Availability

The data underlying this article will be shared on reasonable request to the corresponding author.

## References

[1] Zonozi R , Cortazar FB, Jeyabalan A et al Maintenance of remission of ANCA vasculitis by rituximab based on B cell repopulation versus serological flare: a randomised trial. Ann Rheum Dis 2024;83:351–9. 38123922 10.1136/ard-2023-224489

[2] Zonozi R , Walters LC, Shulkin A et al T cell responses to SARS-CoV-2 infection and vaccination are elevated in B cell deficiency and reduce risk of severe COVID-19. Sci Transl Med 2023;15:eadh4529. 38019932 10.1126/scitranslmed.adh4529 PMC12949954

[3] COVID-19 treatment guidelines. Clinical spectrum. https://www.covid19treatmentguidelines.nih.gov/overview/clinical-spectrum/ (23 July 2024, date last accessed).

[4] Zimmerman O , Altman Doss AM, Ying B et al Immunoglobulin replacement products protect against SARS-CoV-2 infection in vivo despite poor neutralizing activity. JCI Insight 2024;9:e176359. 10.1172/jci.insight.176359 PMC10967375 38175703

[5] Upasani V , Townsend K, Wu MY et al Commercial immunoglobulin products contain neutralizing antibodies against severe acute respiratory syndrome coronavirus 2 spike protein. Clin Infect Dis 2023;77:950–60. 37338118 10.1093/cid/ciad368 PMC10552578

