# Supplementary material for: Protracted COVID-19 pneumonia in B-cell-depleted patients
Source: Rheumatology (Oxford). 2024 Dec 23;64(4):2303–5. doi: 10.1093/rheumatology/keae703 (PMC11962908; doi:10.1093/rheumatology/keae703)
Supplement: keae703_Supplementary_Data [file keae703_supplementary_data.docx]

**Protracted COVID-19 Pneumonia in B Cell Depleted Patients**

**Supplementary Materials**

1. **Concise methods**
2. **Figure S1. Computed tomography images of the patients with protracted COVID-19 for whom the diagnoses were secured only after testing lower respiratory tract samples or serial nasal swabs and anti-SARS-CoV-2 antibody levels at the time of COVID-19 diagnosis and repeat levels after Ig replacement.**

**Concise Methods**

**Rituximab treatment of autoimmune diseases and B cell depletion**

Rituximab or other anti-CD20 treatment of autoimmune diseases in our center typically consists of two 1,000-mg intravenous (IV) doses separated by 2 - 4 weeks for induction, followed by a single 1,000-mg IV dose every 4 - 6 months to start for maintenance. Rituximab intervals were often extended to allow B cell repopulation before redosing when rituximab is continued beyond two years. Peripheral B cell (CD19^+^CD20^+^) counts were typically measured before each rituximab dose or at 3-6 month intervals after stopping rituximab. Partial and full B cell depletion was defined as peripheral CD19^+^CD20^+^ B cells >5 to <100/µL and <5/µL, respectively.

**COVID-19 diagnosis and symptom severity**

COVID-19 was diagnosed by either polymerase chain reaction (PCR) or antigen-based testing at home or hospital. All cases who were identified to have documented COVID-19 were included. Patients who lacked sufficient follow-up data were then excluded. The COVID-19 severity was categorized per the National Institute of Health Clinical Spectrum of SARS-CoV-2 Infection, last updated February 29, 2024.^1^ Protracted COVID-19 pneumonia was defined as worsening disease per symptoms, oxygenation, and/or imaging at or after the 30-day mark since diagnosis.

**Anti-SARS-CoV-2 antibody testing**

Anti-SARS-CoV-2 antibody levels determined at the Massachusetts General Hospital Core Laboratory, from before and after IVIG treatments, were available in 6 patients. The Roche Elecsys anti-spike protein immunoglobulin G (IgG) assay was used to measure anti-spike protein antibodies quantitatively.^2^ The Roche Elecsys Anti-SARS-CoV-2 Total Antibodies test was used to detect antibodies from an immune response to SARS-CoV-2 infection as opposed to vaccinations.^3^ The Total Antibodies assay uses a recombinant protein representing the nucleocapsid antigen to determine antibodies against SARS-CoV-2.

**Treatment of COVID-19**

Treatments for COVID-19 included nirmatrelvir/ritonavir, molnupiravir, remdesivir, steroids, IVIG or tocilizumab. Nirmatrelvir/ritonavir treatment included oral nirmatrelvir 300 mg with ritonavir 100 mg twice daily for 5 days. Remdesivir was mostly given as an IV infusion at 200 mg on day 1, followed by 100 mg daily for 3 days but was extended to 5-20 days for patients with persistent disease. Only one patient received molnupiravir, which included 800 mg every 12 hours for 5 days. IVIG was usually given at 0.4 g/kg/day for 3 to 5 days. Steroid treatments included prednisone > 20 mg or equivalent for at least 3 days and often included dexamethasone 6 mg once daily for up to 10 days. The doses of all medications were adjusted for kidney function, liver function, and weight based on manufacturers’ suggestions.

**References**

1. Clinical Spectrum. COVID-19 Treatment Guidelines. Accessed May 20, 2024. https://www.covid19treatmentguidelines.nih.gov/overview/clinical-spectrum/

2. Elecsys® Anti-SARS-CoV-2 S. Diagnostics. Accessed July 23, 2024. https://diagnostics.roche.com/global/en/products/params/elecsys-anti-sars-cov-2-s.html

3. Elecsys® Anti-SARS-CoV-2. Diagnostics. Accessed July 23, 2024. https://diagnostics.roche.com/global/en/products/params/elecsys-anti-sars-cov-2.html


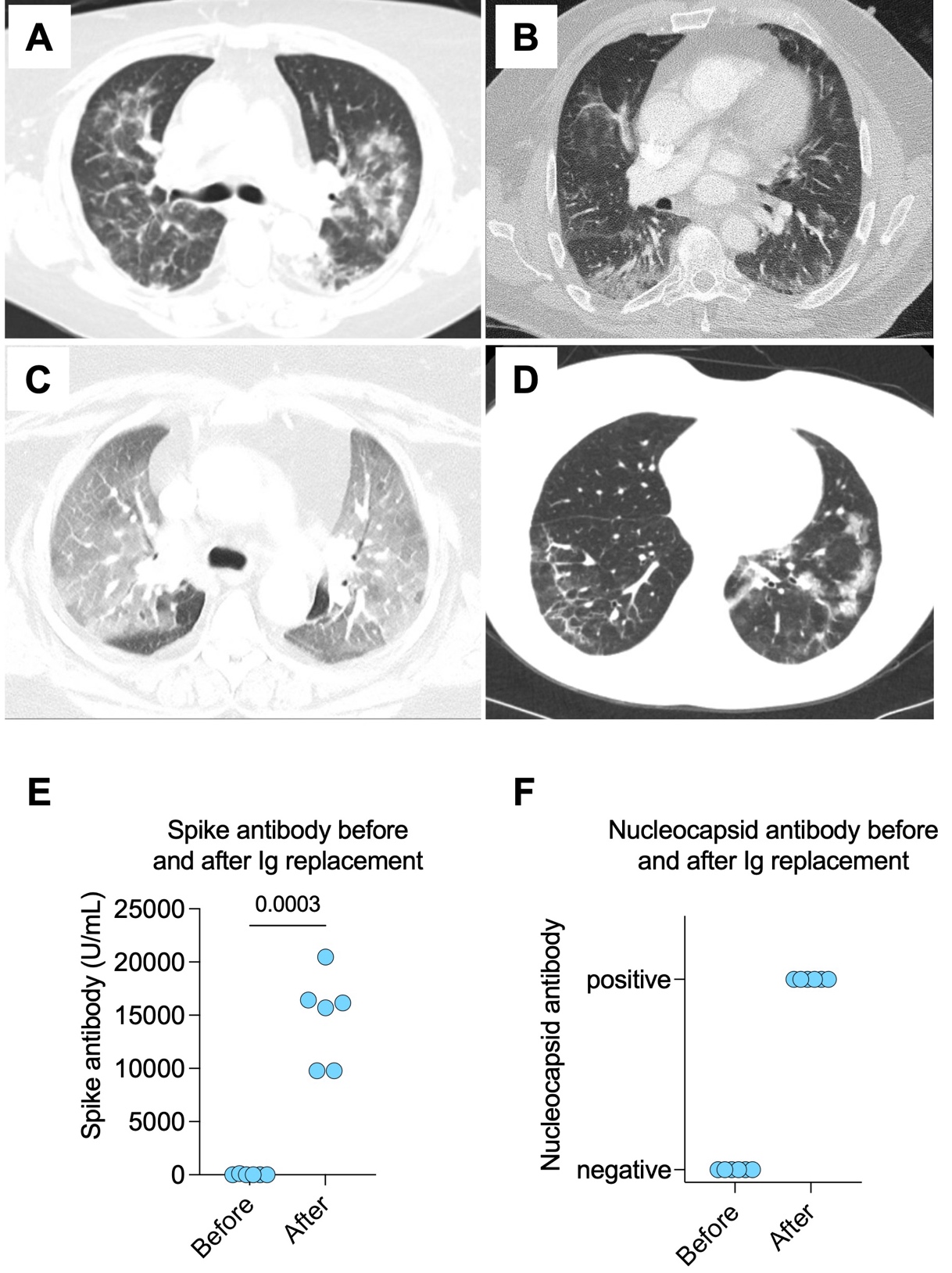


**Figure S1. Computed tomography images of the patients with protracted COVID-19 for whom the diagnoses were secured only after testing lower respiratory tract samples or serial nasal swabs and anti-SARS-CoV-2 antibody levels at the time of COVID-19 diagnosis and repeat levels after Ig replacement.** A) Computed tomography (CT) image of the typical patient outlined in the letter who developed protracted COVID-19 and was diagnosed only after PCR testing of bronchoalveolar lavage at day 35. B-C) CT images of two patients with protracted COVID-19 for whom the diagnoses were secured only after PCR testing of sputum at days 70 and 39, respectively. D) CT image of a case in which the COVID-19 diagnosis was only secured after serial nasal swabs (>5) at day 59 since symptom onset. Treatments led to rapid clinical improvement in all. E) Paired anti-spike and F) nucleocapsid antibody levels of individual patients before and after Ig replacement (n=6). Paired t-test is used for before and after comparison.
